# ‘Act on Oncology’ as a New Comprehensive Approach to Assess Prostate Cancer Centres – Method Description and Results of a Pilot Study

**DOI:** 10.1371/journal.pone.0106743

**Published:** 2014-09-05

**Authors:** Wieland Voigt, Josef Hoellthaler, Tiziana Magnani, Vito Corrao, Riccardo Valdagni

**Affiliations:** 1 Siemens AG, Healthcare Sector, Customer Solutions Division, H CX CRM-VA HCC ONC, Erlangen, Germany; 2 Fondazione IRCCS Istituto Nazionale dei Tumori, Milan, Italy; ISPO, Italy

## Abstract

**Background:**

Multidisciplinary care of prostate cancer is increasingly offered in specialised cancer centres. It requires the optimisation of medical and operational processes and the integration of the different medical and non-medical stakeholders.

**Objective:**

To develop a standardised operational process assessment tool basing on the capability maturity model integration (CMMI) able to implement multidisciplinary care and improve process quality and efficiency.

**Design, Setting, and Participants:**

Information for model development was derived from medical experts, clinical guidelines, best practice elements of renowned cancer centres, and scientific literature. Data were organised in a hierarchically structured model, consisting of 5 categories, 30 key process areas, 172 requirements, and more than 1500 criteria. Compliance with requirements was assessed through structured on-site surveys covering all relevant clinical and management processes. Comparison with best practice standards allowed to recommend improvements. ‘Act On Oncology’(AoO) was applied in a pilot study on a prostate cancer unit in Europe.

**Results and Limitations:**

Several best practice elements such as multidisciplinary clinics or advanced organisational measures for patient scheduling were observed. Substantial opportunities were found in other areas such as centre management and infrastructure. As first improvements the evaluated centre administration described and formalised the organisation of the prostate cancer unit with defined personnel assignments and clinical activities and a formal agreement is being worked on to have structured access to First-Aid Posts.

**Conclusions:**

In the pilot study, the AoO approach was feasible to identify opportunities for process improvements. Measures were derived that might increase the operational process quality and efficiency.

## Introduction

Owing to the growing options for diagnosis and treatment, the optimal management of prostate cancer patients is still controversial. Treatment options include different kinds of surgery and radiation therapies as well as hormonal therapy, chemotherapy, and immunotherapy. These options are supplemented by observational approaches such as active surveillance and watchful waiting [1], [2]. Since some therapies seem to be equally effective, patients need to be informed objectively about the risks and benefits of each option [3], [4], [5].

A multidisciplinary approach facilitates the shared decision-making between patients and specialists. Multidisciplinary disease management is further associated with improved outcome in breast cancer, prostate cancer as well as other types of cancer [6], [7], [8], [9], [10], [11], [12]. Additionally, it seems to positively impact on both cost-effectiveness of care and patient satisfaction [10], [13]. Multidisciplinary management, however, requires well-integrated medical and management services and an effective coordination of clinics, diagnostic routines, treatments, follow-ups, and appointments with medical and non-medical experts [14], [15]. These patient care processes have to comply with operational processes, appropriate human, technical, and financial resources, and optimal infrastructural, administrative, and management support [10], [15].

Earlier quality assessments indicated that the quality of cancer care varies among prostate cancer centres [16]. Quality assessment and improvement initiatives as well as certification programs have therefore been developed to enforce medical standards, adherence to guidelines, and to strengthen the multidisciplinary approach [15], [17]. These programs mainly focus on clinical and outcome related parameters. However, quality and efficiency of operational processes and centre infrastructure as well as the functional integration of services are taken less into account. In addition, maintenance and improvement of care quality by, for example, certification programs add to operational costs, resulting in a steadily increasing economic pressure [17]. Given that organisational improvements and streamlined clinical processes were shown to reduce costs while retaining quality, process modelling might be an effective tool to counteract economic constraints [17], [18], [19].

In a continuous improvement concept for complex medical organizations, the assessment of both quality and efficiency of medical and non-medical processes as well as the definition of relevant performance indicators are required [20], [21], [22]. Recognizing this need, we adapted the ‘capability maturity model integration’ (CMMI) approach to assess complex medical organizations like cancer centers [23]. Basically, CMMI aims at improving the quality and efficiency of processes as well as the integration of organisational components and establishes maturity levels for each individual process [24]. These levels can be used to benchmark processes and organisations.

Recently, the CMMI approach has been successfully applied by our group to improve processes in radiology departments [25]. Here we describe the application of the CMMI to more complex medical organisations such as prostate cancer centres. Model development comprised the collection of information on all relevant aspects of cancer centres and structuring these into categories, key process areas, requirements, and criteria. According to the CMMI principle, a hierarchically structured systematic assessment matrix was created that enables on-site surveys at cancer centres. Comparison with current best practice standards then provides the basis to recommend applicable solutions. A pilot study at a prostate cancer centre in Italy identified several measures for process improvement and, importantly, proved the general applicability of our approach.

## Materials and Methods

### Project members

The Act On Oncology approach for the systematic assessment of prostate cancer centres was developed by four consistent team members with a medical or consulting background (core team). For specific issues, the core team was supplemented by associate members with medical (2), consulting (2), biomedical (1), or technical background (2).

### Act On Oncology database

To develop the model, scientific literature, certification criteria of the German cancer society [26], and medical guidelines were analysed and consolidated [1], [27]. The model was enriched by best practice elements, which were derived from workshops with international experts and from best practice experiences of both renowned cancer centres worldwide and patient organisations (Germany, USA). Best practice experience also came from the professional background of team members in internal medicine, radiation therapy, palliative care, intensive care, and medical oncology as well as from the knowledge gathered during more than 500 hospital consulting projects worldwide. Each piece of information was discussed and prioritised in regular core team meetings.

### Development of the organisational structure

As an established tool for continuous process optimisation in the industry, the CMMI method provided the basis for the Act On Oncology approach. It defines five maturity levels of processes: Level 1 (initial; situational treatment) is defined by rather unmanaged and arbitrary operational procedures; level 2 (repeatable) is characterised by the use of guidelines, leading to controlled processes; level 3 (defined) uses well-characterised operational procedures and defined standard processes; level 4 (managed) applies quantitative key performance indicators for the management of processes, making process outcomes more predictable; level 5 (optimised) involves the continuous improvement of processes by the use of key performance indicators [24], [28].

**Figure 1:**
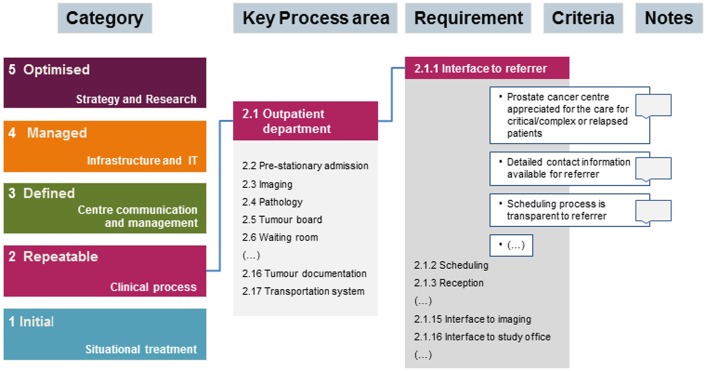
Illustration of the hierarchically structured Act On Oncology model. Five categories derived from the Act On Oncology database were assorted to different maturity levels according to CMMI (left). The figure exemplifies the ramification of the key process area ‘outpatient department’ into requirements and criteria, which are further substantiated by notes. With more than 1500 criteria, the model describes best practice elements in all different fields of a prostate cancer centre.

To adjust the CMMI methodology [24], [28] organisational categories were defined and assorted to different process levels, based on their relevance for process characterisation and development. For each category, key process areas were defined and partitioned into requirements for prostate cancer centres. These requirements were specified by criteria, which, in turn, were substantiated by best practice elements extracted from the sources mentioned above (Figure 1). To continuously improve our approach, the developmental progress was subjected to periodic reviews by team members. Mindjet Mindmanager Software was applied to manage the resulting hierarchically structured knowledge database.

### Structured survey and systematic assessment

A structured survey matrix was derived from the comprehensive database by using the Mindjet Mindmanager Software. This survey matrix is thought to be used in on-site interviews with variable numbers of stakeholders depending on the size and organisation of a centre. Interviewees might comprise physicians, executive management, medical and non-medical personnel, administrational staff, and referrers. Obtained information is then entered into an EXCEL spreadsheet database to allow a standardised and systematic evaluation of each requirement, based on the fulfilment of each criterion. Next, fulfilment is assessed by the team with best practice standards as reference (as defined by the criteria and notes) and subsequently ranked on a 4-point Likert-scale ranging from 1 (not fulfilled) to 4 (completely fulfilled). If information cannot be evaluated, fulfilment is rated as ‘0’. Data are summarised in radial bar charts, along with a description of problems and potential solutions. In general, Act On Oncology comprises three phases (systematic assessment, elaboration of results, presentation of findings). The on-site assessment by three consultants usually lasts for 3 to 4 days. The elaboration and presentation of results requires 2 to 3 weeks.

### Pilot study and validation phase

In December 2012, a pilot study was conducted in a specialised prostate cancer unit at Fondazione IRCCS Istituto Nazionale dei Tumori (INT) in Milan, Italy. The INT was selected because of its long-term experiences in both multidisciplinary care of prostate cancer patients and prostate cancer research [13], [15]. The multidisciplinary clinic at INT has two components, weekly multidisciplinary consultations of specialists and weekly clinical case discussions.

During the on-site survey, 24 employees representing all relevant clinical processes including management, controlling, data management, and clinical trial handling were interviewed by two members of the core team and one associate member, accompanied by a translator if necessary (Tab. 1). The duration of each interview was about one hour; the whole interview phase lasted three days. Additional information on infrastructure and IT-related subjects was acquired during a guided tour through the facility. Obtained information was analysed as described above and presented in a standardised presentation that included results and recommendations.

**Table 1:** Listing of interview partner.

Affiliation of Interview partner	Function of Interview partner
Program Management	Director of Prostate Cancer Program, Coordinator of patient scheduling, Project Manager, Controlling, Patient Secretary, Data manager, IT-specialist
Radiation therapy	2x Radiation Oncologist (dedicated to Brachytherapy), 2x Radiation Oncologist (dedicated to External Radiotherapy), Coordinator Radiotherapy, Medical Physicist, Nurse
Urology	Urologist
Medical Oncology	2x Medical Oncologist
Radiology	Director of Radiology, Radiologist
Clinical trials	Research nurse, Trial physician
Supportive Therapy	Psycho Oncologist, Palliative Care/Pain specialist, Supportive care specialist

## Results

### Act On Oncology Model

The following five categories were identified as the superordinate topic areas that characterise the organisational structure of prostate cancer centres: ‘clinical processes’, ‘centre communication’, ‘centre management’, ‘infrastructure and IT’, and ‘strategy and research’. Corresponding to the CMMI approach, categories were assorted to maturity levels (situational treatment  =  1; clinical process  =  2; centre communication and management  =  3; infrastructure and IT  =  4; strategy and research  =  5). The five categories were then split into 30 clusters of related activities (key process areas), which were thought to best characterise the main functional units. In detail, 16 key process areas were defined for clinical processes, 8 for centre communication and management, 4 for infrastructure and IT, 2 for strategy and research. The selection of process areas was continuously challenged and revised if necessary. Next, these 30 key process areas were divided into 172 requirements. To finally characterise each requirement, 1500 criteria were defined and further substantiated by notes (Figure 1).

**Figure 2:**
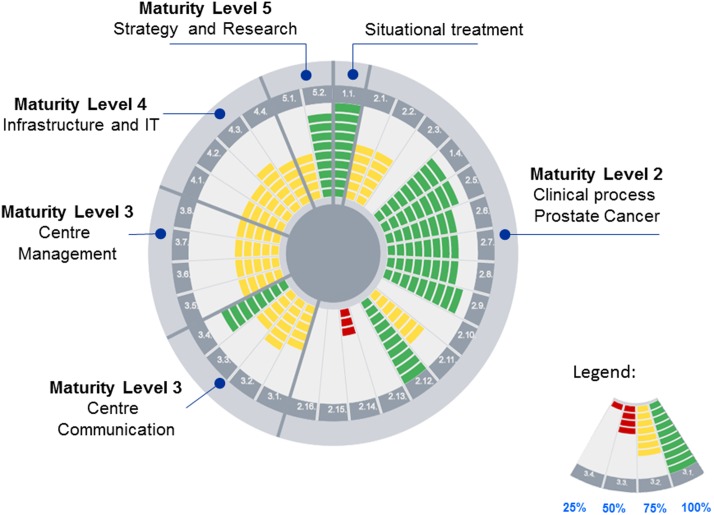
Example of an automatically generated radial bar chart providing an overview of the level of fulfilment of each key process area. Beginning at the centre point, the level of fulfilment of key process areas is indicated along the radius in steps of 10%. Green colour indicates fulfilment of up to 100% (red ≤ 50%, yellow ≤ 75%). On the outer circle, the corresponding maturity level of each category is provided.

Having defined and organised the different categories, process areas, requirements, and criteria, a structured survey matrix for on-site interviews was created. After the interview phase, obtained information was entered into a spreadsheet database and the degree of fulfilment of each requirement was appraised and quantified by the team. Results were then visualised in radial bar charts, thus immediately giving an overview of the process quality of the whole centre. A three-colour code illustrated the urgency of management attention (Figure 2).

### Pilot study

During the on-site assessment of the INT, several processes and features were considered best practice when compared with the best practice experience integrated in the Act On Oncology database. These processes and features included the weekly multidisciplinary clinics, the weekly multidisciplinary case discussions, the psychological support for patients early in the decision making process, and the existence of active surveillance protocols. In the multidisciplinary clinics, prostate cancer patients are counselled by a multidisciplinary team consisting of urologists, radiation oncologists, medical oncologists (for advanced, hormone-refractory and metastatic disease), and psycho-oncologists. Supportive care, rehabilitation, and specialist palliative care interventions are available on demand. Case discussions then aim at sharing decisions made in the multidisciplinary clinic, tailoring therapeutic strategies, and evaluating the adherence to guidelines. Urologists, radiation oncologists, medical oncologists, psychologists regularly participate in these discussions, while pathologists, radiologists, and experts in supportive and palliative care join in on demand. Trained administrative personnel is further employed to improve the clinical workflow by reminding patients of their clinic appointments and collecting required information.

Together with the preparation and adoption of shared institutional guidelines, these measures were considered to increase the quality of care and to contribute to a successful enrolment in protocols, above all in active surveillance studies. Regular patient satisfaction surveys, the high level of evidence based decision making, and the considerable contribution to clinical research were viewed as additional quality indicators.

In contrast, substantial need for improvements was identified in centre management and infrastructure. For example, the collaboration between the departments of urology and radiation oncology was not defined at the management and strategy level, and the corporate identity within the prostate cancer unit was not particularly evident. Most importantly, the prostate cancer program received limited support from the hospital administration. In particular, there was no sufficient budget and staffing allocated to the program, resulting in a continuous need for other non-institutional funding sources.

**Figure 3:**
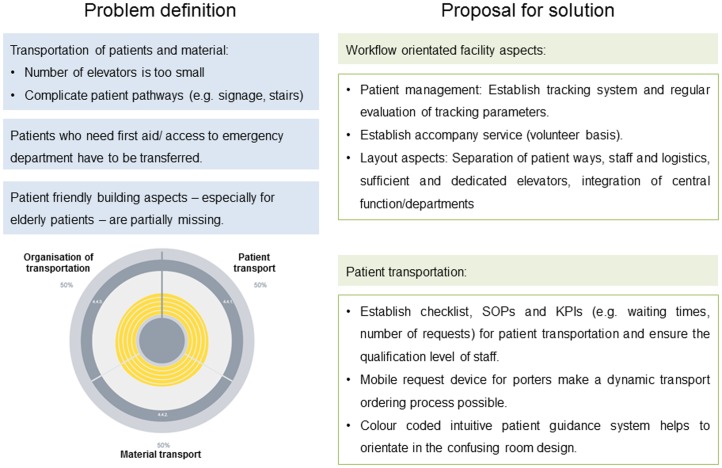
Exemplary result of the pilot study. The graph (bottom left) shows a 50% fulfilment of requirements related to organisation of transportation, patient transport, and material transport, along with a description of problems and potential solutions (SOP: standard operating procedure; KPI: key performance indicator).

Moreover, the INT infrastructure did not support the clinical workflow optimally. Several bottlenecks such as insufficient elevator capacity, confusing patient walkways, the absence of an emergency room, and the lack of formal agreements to rule specialists’ collaborations were identified. In addition, the facility did not fulfil the requirements of a patient-friendly building (in particular, elderly/disabled patients). Problems identified within the key process area ‘patient transportation, material transportation, and organisation of transportation’ along with their potential solutions are exemplary illustrated in Figure 3.

In summary, beside several best practice elements, some opportunities for improvements of operational processes, management, and infrastructure were identified. Concrete measures were suggested in a systematic management summary. A reassessment is planned in about two years to evaluate whether the suggested measures lead to the expected improvements.

## Discussion

Multidisciplinary care has been recognised to improve the quality of care and to positively influence outcome in some types of cancer such as breast cancer [6], [12], [29]. Therefore, multidisciplinary management of patients with other cancers like prostate cancer is increasingly advocated [10], [13], [14], [30]. Multidisciplinary management in specialised prostate cancer centres implies new organisational and management challenges [10], [13], [14]. Hospitals traditionally have a vertical management structure with individually operated clinics or departments, while a horizontal management approach would be more appropriate to align and integrate the different medical, supportive, and management functions to achieve high medical and operational standards [21], [22], [31].

To improve the standard of cancer care, different national and European certification programs for breast and prostate cancer care have been launched representing a promising step towards continuous quality improvement and standardisation of cancer care [15], [17], [29], [32], [33]. However, quality and efficiency of operational processes and the level of integration of services are usually not in the main focus of certification programs. Thus, to complement existing certification programs, we developed the Act On Oncology approach which isas to our knowledge the first example to apply the CMMI for the structured assessment of prostate cancer centers. In contrast to other consulting practices in the market, the predefined interview matrix of Act On Oncology allows a quick and reproducible analysis of a center within a few days.

Act On Oncology aims to generate a holistic view on the operational processes in each unit of a cancer centre, their interfaces to each other, and the level of integration. Therefore, the categories ‘centre management’ and ‘communication’ were specifically addressed. As suggested by recent publications, the transition of a purely vertical organised centre into a cross-linked structure depends on different management functions and an effective communication among departments and divisions [21], [22], [31]. Communication strategies are useful to generate a corporate identity within a centre but also in the outbound communication with patients and referrer. Cross-departmental collaboration is not only a prerequisite to create a corporate strategy, but is also required for the conduct of clinical trials, which are an important source for scientific impact and financial resources. We therefore identified ‘strategy and research’ as another superordinate category of our model.

Healthcare IT and centre infrastructure were identified as further overarching categories since both influence the performance of healthcare delivery [34]. In health care industry, IT is an asset that helps to improve quality of health services, to manage rising costs and changing organisational needs, and to improve data exchange within a centre.

As first of its kind, Act On Oncology establishes reference points to which process quality and efficiency as well as the level of integration of the different stakeholders can be related. Improving the quality of care relies on several factors, from the adoption and routine application of guidelines for diagnosis, treatment and follow-up, to the multidisciplinary management of patients [14], [35], [36]. In addition, quality of care also bases on structural issues such as the provision and integration of different services and resources [13], [15], [21]. Measures to meet these requirements may include the centralisation and standardisation of multidisciplinary care, the application of IT solutions, the improvement of management structures, the streamlining of workflow processes including interfaces with referrers, and the development of joint strategies [10], [13], [14], [21], [22], [34], [37].

In our pilot study, Act On Oncology identified areas for improvement on multiple levels. Despite the promising achievements in the implementation of multidisciplinarity at the staff level, the pilot study revealed the need for an overriding management framework to promote the collaboration among departments, to allow a corporate identity to evolve, and to canvas additional support. In this regard, it is important to note that an advanced organisational infrastructure appears to improve both outcomes of cancer patients [16], [38] and cost efficiency of clinical processes [17], [18], [19], [39]. As a first improvement that has been implemented in the meanwhile, the centre administration and the Health Director at INT described and formalised the organisation of the prostate cancer unit. The document defines both categories of personnel assigned to the prostate cancer unit (core team, non-core team, project team) and clinical activities (multidisciplinary clinics, observational setting clinics, clinical case discussions). The implementation of this suggested measure can be considered as the first positive effect of the Act On Oncology assessment. During the assessment, several features of the prostate cancer unit were ranked as best practice, reflecting the efforts that have already been made at the INT: the multidisciplinarity of designated weekly clinics and tumour board meetings, the presence of a psychologist in the multidisciplinary clinics and case discussion meetings, and the existence of an active surveillance program [10], [11], [13], [14], [15], [40], [41].

Act On Oncology employs a structured and predefined interview and assessment matrix. The experiences from the development phase and the pilot study, along with experiences from the assessment of radiology departments [25], suggest a high level of reproducibility with low interobserver variability. However, one limitation of our method is related to the global diversity of health care systems. Although several cancer centres in Germany, Italy, and the US have been analysed, this diversity might not be appropriately addressed by our model. This is particularly important once our model should be used for global benchmarking of cancer centres. To address this limitation, our approach is subjected to a continuous improvement process. We expect that, after additional global assessments, Act On Oncology might be applied as a benchmarking tool in the future [22].

## Conclusion

In conclusion, Act On Oncology provides a feasible tool to evaluate quality and efficiency of operational processes in prostate cancer centres. During a pilot study, several best practices and opportunities could be identified. Measures for improvement were elaborated and their effectiveness has to be proven in a future reassessment. Broad scale assessments will be necessary to apply Act On Oncology as a benchmarking tool for cancer centres.
